# Nordihydroguaiaretic Acid: From Herbal Medicine to Clinical Development for Cancer and Chronic Diseases

**DOI:** 10.3389/fphar.2020.00151

**Published:** 2020-02-28

**Authors:** Gina Manda, Ana I. Rojo, Elena Martínez-Klimova, José Pedraza-Chaverri, Antonio Cuadrado

**Affiliations:** ^1^ Department Cellular and Molecular Medicine, Victor Babes National Institute of Pathology, Bucharest, Romania; ^2^ Department of Biochemistry, Faculty of Medicine, Autonomous University of Madrid, Centro de Investigación Biomédica en Red sobre Enfermedades Neurodegenerativas (CIBERNED), Instituto de Investigación Sanitaria la Paz (idiPAZ), Instituto de Investigaciones Biomédicas “Alberto Sols” UAM-CSIC, Madrid, Spain; ^3^ Department of Biology, Faculty of Chemistry, National Autonomous University of Mexico (UNAM), Mexico City, Mexico

**Keywords:** catechol, quinone, electrophiles, NRF2, KEAP1, cytoprotection, oxidative stress, inflammation

## Abstract

Nordihydroguaiaretic acid (NDGA) is a phenolic lignan obtained from *Larrea tridentata*, the creosote bush found in Mexico and USA deserts, that has been used in traditional medicine for the treatment of numerous diseases such as cancer, renal, cardiovascular, immunological, and neurological disorders, and even aging. NDGA presents two catechol rings that confer a very potent antioxidant activity by scavenging oxygen free radicals and this may explain part of its therapeutic action. Additional effects include inhibition of lipoxygenases (LOXs) and activation of signaling pathways that impinge on the transcription factor Nuclear Factor Erythroid 2-related Factor (NRF2). On the other hand, the oxidation of the catechols to the corresponding quinones my elicit alterations in proteins and DNA that raise safety concerns. This review describes the current knowledge on NDGA, its targets and side effects, and its synthetic analogs as promising therapeutic agents, highlighting their mechanism of action and clinical projection towards therapy of neurodegenerative, liver, and kidney disease, as well as cancer.

## Introduction

Nordihydroguaiaretic acid (NDGA), also called masoprocol {IUPAC name: 4-[4-(3,4-dihydroxyphenyl)-2,3-dimethylbutyl]benzene-1,2-diol}, is a phenolic lignan mainly extracted from the five plant species that constitute the genus *Larrea* (Arteaga et al., 2005; Peralta et al., 2018). A general source of NDGA is the leaves of *Larrea tridentata*, also known as “chaparral”, “creosote bush”, and “gobernadora”, which is abundant in the deserts of Mexico and southwest USA (Arteaga et al., 2005). NDGA accounts for approximately 10% of the leaves’ dry weight of *L. tridentata* and 80% of all flavonoids and lignans that are found in the resin of this plant (Floriano-Sanchez et al., 2006).

The leaves have been used in traditional medicine of the mentioned regions for the treatment of over 50 diseases, including rheumatism, arthritis, diabetes, pain, and inflammation (Arteaga et al., 2005). More recently, NDGA started to be tested in other pathologies that are becoming prevalent as a result of population aging (Sadagurski et al., 2017). NDGA has been utilized as an antioxidant food preservative and as nutritional supplement, mainly in the form of chaparral tea. The best characterized effects of NDGA are: 1) the ROS scavenging nature of NDGA decreases the pro-oxidant effects of inflammation; 2) the inhibitory effects on lipoxygenases (LOX) activity, leading to the reduction of lipid hydroperoxides (5-HEPE and 5-HETE at 50 µM NDGA) that trigger oxidative stress due to their decomposition to free radicals (Mashima and Okuyama, 2015) the induction of ROS production through activation of NADPH-oxidases, MAPKs, etc. (Li Q. et al., 2016; Nagahora et al., 2017); 3) the activation of endogenous antioxidant responses mediated by NRF2. Most of the pleiotropic effects that have been attributed to this compound are briefly summarized in **Table 1**.

**Table 1:** Some NDGA targets. The table summarizes some of the best characterized NDGA targets.

Molecular target	NDGA effect	References
5-LOX, 12-LOX, 15-LOX	Inhibition	(Tateson et al., 1988; Pavani et al., 1994; Vasquez-Martinez et al., 2007)
Lipoprotein lipase	Inhibition	(Kang et al., 2019)
Reactive oxygen species	Scavenging	(Floriano-Sanchez et al., 2006)
α-amylase, α-glucosidase and dipeptidyl peptidase 4	Inhibition	(Roskar et al., 2016)
mTORC1	Inhibition	(Zhang et al., 2012)
large conductance Ca^2+^-activated K^+^	activation	(Yamamura et al., 2002)
KEAP1	KEAP1 inhibition/NRF2 activation	(Satoh et al., 2008; Rojo et al., 2012)
Insulin-like receptor-1 (Tyr kinase receptor)	Inhibition	(Youngren et al., 2005)
c-ErbB2/HER2/Neu (Tyr kinase receptor)	Inhibition	(Youngren et al., 2005; Rowe et al., 2008)
Transforming growth factor β type 1 receptor (Ser/Thr kinase receptor)	Inhibition	(Youngren et al., 2005; Li et al., 2009)
GSH	depletion	(Im and Han, 2007)
PTEN (Redox-sensitive phosphatase)	Inhibition	(Rojo et al., 2014)
DUSPs (Redox-sensitive phosphatase)	Inhibition	(Liu et al., 2012; Rios et al., 2014)

PTEN and DUSPs are postulated as NDGA targets based on information from other polyphenols (see text).

Despite the existence of many preclinical studies that highlight the therapeutic potential of NDGA, the fact is that most of its beneficial effects are not supported by clinical studies, as it usually happens with parapharmaceutical products (Abou-Gazar et al., 2004; Arteaga et al., 2005). Moreover, it was found that excessive consumption of this phytochemical may damage several organs including kidney and liver (Goodman et al., 1970; Evan and Gardner, 1979), hence raising awareness about the need of careful control of NDGA dosing and treatment length. Therefore, the clinical development of NDGA and its analogs is progressing slowly. This review will critically discuss the best characterized mechanisms and targets attributed to NDGA, safety concerns and the potential of NDAG analogs for clinical translation.

## Antioxidant and Electrophilic Activities of NDGA

**Figure 1:**
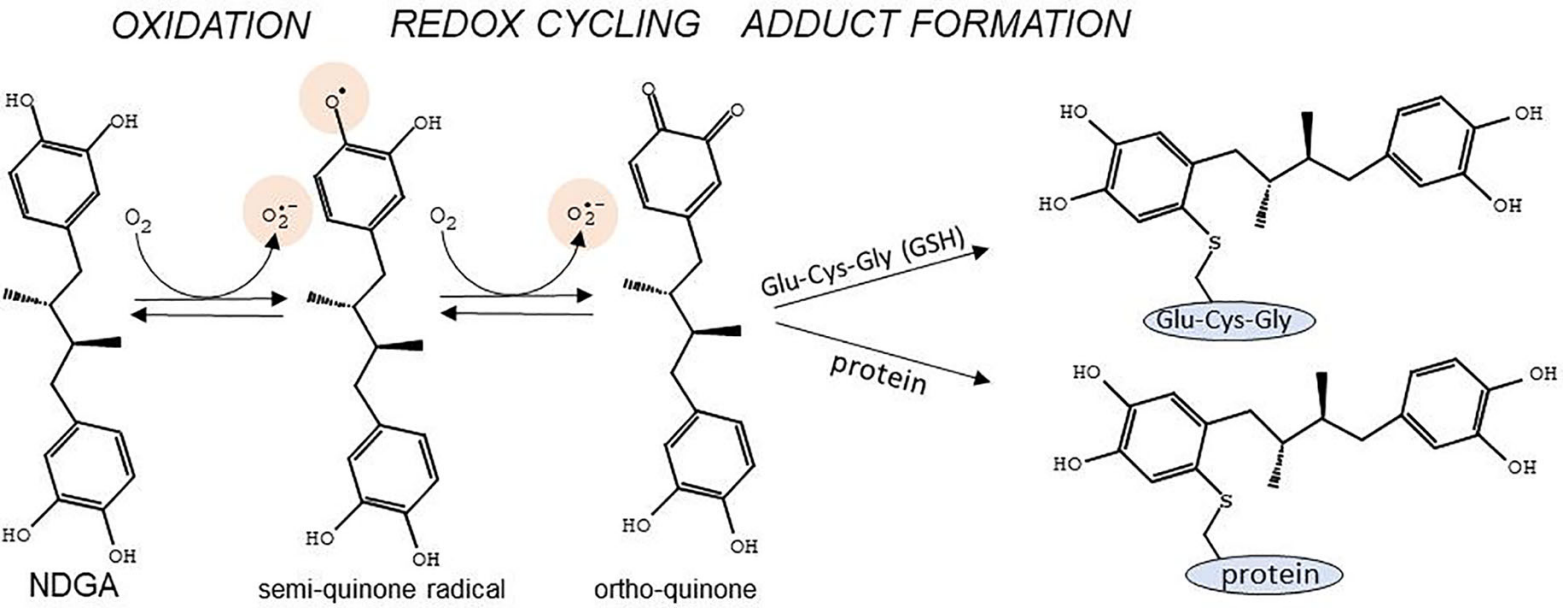
Main reactions for conversion of the catechol rings of NDGA into *semi*-quinone and *ortho*-quinone, and adduct formation with cysteines in glutathione (Glu-Cys-Gly) or in proteins. Of note are the generation of the *semi*-quinone free radical as well as superoxide anion during the redox cycling reactions. Adapted with permission from (Billinsky et al., 2007). Copyright (2007) American Chemical Society.

NDGA presents two catechol rings that confer both cytoprotective and cytotoxic effects depending on the dosage and the context. The cytoprotective effect stems from the strong scavenging activity of NDGA against multiple types of Reactive Oxygen Species (ROS) such as peroxynitrite, singlet oxygen, hydroxyl radical, superoxide anion, and hypochlorous acid (Floriano-Sanchez et al., 2006). NDGA can donate one electron and one proton from each of its four hydroxyl groups contained in the two catechol rings, converting itself into an oxidized catechol-quinone (Yam-Canul et al., 2008). Since NDGA is a symmetrical molecule with two catechol groups, both catechols can be oxidized to quinones. The reactions involved in the oxidative modifications of NDGA have been described previously (Billinsky et al., 2007; Billinsky and Krol, 2008) (**Figure 1**). Briefly, at physiological pH, NDGA rapidly auto-oxidizes, resulting in the formation of a *semi*-quinone radical which is further converted in a second oxidation step to generate *ortho*-quinone and superoxide anion, mostly spontaneously or through peroxidases- and cytochrome p450-catalyzed reactions (Billinsky and Krol, 2008). *Ortho*-quinone may be converted back to the reactive *semi*-quinone by cellular NADPH-dependent reductases (O’brien, 1991; Monks et al., 1992; Chichirau et al., 2005). This redox cycle is highly toxic, as it evokes superoxide-generated oxidative stress, which may become deleterious at high concentrations of NDGA. The *ortho*-quinone is a highly electrophilic Michael reaction acceptor (Talalay et al., 1988) that reacts with sulfur neutrophiles such as cysteine residues in glutathione (GSH) and in various proteins (Powis, 1987). Adduct formation of *ortho*-quinone with GSH results in increased excretion of these compounds, but at the cost of depleting the GSH pool, hence leading to a shift in the redox balance towards deleterious oxidative stress. On the other hand, adduct formation with cysteines in critical proteins may lead to changes in signaling pathways that trigger cytoprotective mechanisms through NRF2 activation (**Figure 1**).

Therefore, the effect of NDGA on cell physiology depends on the balance between its protective effect, which is mediated by its antioxidant nature and electrophilic interaction with several signaling proteins, and its toxic effect, which is related to enhanced superoxide formation and GSH depletion. This balance is narrowly dependent on NDGA concentration. Although not analyzed for NDGA, diphenols provide cytoprotection at low doses, whilst being toxic at high doses (Satoh et al., 2013). In this context, NDGA has a redox potential and a geometric distribution of atoms that make it suitable for interaction with cysteines in proteins over a small range of concentrations at which GSH levels are not substantially depleted. Moreover, NRF2 activation by NDGA (described in detail later in this review) leads to increased expression of the two subunits that conform the glutamate-cysteine ligase (GCLC and GCLM) which is the rate-limiting enzyme in GSH biosynthesis, hence contributing to the maintenance of the cellular GSH pool.

## NDGA Is a Pan-Lipoxygenase Inhibitor

**Figure 2:**
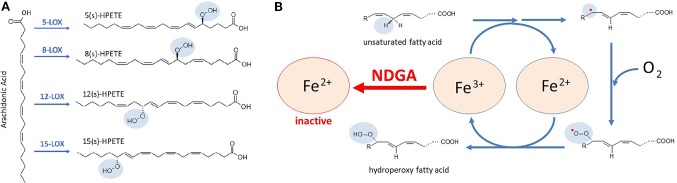
Catalytic mechanism proposed for lipoxygenases (LOXs) inhibition by NDGA. **(A)** conversion of arachidonic acid to HPETEs by specific LOX enzymes that are targeted by NDGA. **(B)** LOXs contain a non-heme Fe in the catalytic center that allows redox cycling of the enzyme, coupled with oxygen consumption. In the first reactions, unsaturated fatty acids, such as arachidonic acid, undergo a hydrogen abstraction and electron rearrangement, converting Fe^3+^ to Fe^2+^, and yielding a free radical (red dot). Then, a molecule of oxygen is taken to form a peroxy radical. Finally, the conversion of peroxy radical to hydroperoxy fatty acid is coupled to the regeneration of Fe^3+^. NDGA maintains Fe in the Fe^2+^ form, hence breaking the redox cycle of LOXs and resulting in its inactivation.

Lipoxygenases (LOXs) are non-heme iron-containing enzymes (six isoforms have been identified in humans) that catalyze the stereospecific oxygenation of *cis*,*cis*-1,4-pentadiene moieties of polyunsaturated fatty acids (PUFAs), such as arachidonic acid, eicosapentaenoic acid and docosahexaenoic acid, and formation of their corresponding hydroperoxy-derivatives, which may be further reduced by glutathione peroxidases. For instance, LOXs catalyze the formation of hydroperoxyeicosatetraenoic acids (HPETEs) from arachidonic acid (**Figure 2A**). HPETEs are subsequently reduced and transformed into bioactive eicosanoids such as 5-hydroxyeicosatetraenoic acid (5-HETE) and 5-hydroxyeicosapentaenoic acid (5-HEPE) which can be further metabolized to hepoxilins, lipoxins, and resolvins. These metabolites are versatile signaling molecules that play an important role in many physiological and pathological processes. Of utmost importance for the immune response is the 5-LOX-mediated generation of leukotriene A4 (LTA4) and its further transformation, by LTA4 hydrolase, into the pro-inflammatory leukotriene B4 (LTB4). LTB4 regulates inflammatory pathways and immune responses against infection and tissue injury (Brandt and Serezani, 2017).

LOXs were the first identified molecular targets of NDGA (Tang et al., 1996; Tang and Honn, 1997; Tong et al., 2002; Floriano-Sanchez et al., 2006; Czapski et al., 2012), which was initially defined as a pan-LOX inhibitor with micromolar and sub-micromolar IC_50_ values (**Table 2**). Accordingly, the biologic effects of NDGA were firstly explained from the LOX inhibition perspective in various pathologic conditions. For catalysis, the iron component of the LOX enzymes must cycle between Fe^2+^ and Fe^3+^ states (**Figure 2B**) and the potent antioxidant activity of NDGA is halting iron in the Fe^2+^ state (Nelson et al., 1991). As shown in **Table 2**, micromolar and sub-micromolar NDGA concentrations inhibit various LOX isoforms including both 15-LOX-1 and 15-LOX-2. Compared to the FDA approved 5-LOX inhibitor zileuton (IC_50_ = 0.15 µM) (Braeckman et al., 1995), NDGA has a lower IC_50_ value (IC_50_ = 0.097 µM) for human 5-LOX (Estrada-Valencia et al., 2019).

**Table 2:** Inhibitory action of NDGA on lipoxygenases (LOXs).

LOX	IC_50_ (µM)	Extracts from:	References
Arachidonate 5-lipoxygenase (5-LOX)	0.8	Leukocytes	(Tateson et al., 1988)
Arachidonate 12-lipoxygenase (12-LOX)	2.6	SF9 cells transfected with human LOX genes	(Vasquez-Martinez et al., 2007)
Arachidonate 5-lipoxygenase-1 (15-LOX-1)	0.25		
Arachidonate 15-lipoxygenase-2 (15-LOX-2)	0.11		
Arachidonate 12/15-lipoxygenase 15/12-LOX	0.1		
Arachidonate 12-lipoxygenase (12-LOX)	3-5	Human platelets	(Pavani et al., 1994)
Arachidonate 5-lipoxygenase (5-LOX)	2.3	Nucleated platelets	(Chen et al., 2005)
Arachidonate 12-lipoxygenase (12-LOX)	1.6		
Arachidonate 15-lipoxygenase (15-LOX)	1.7		
Arachidonate 5-lipoxygenase (5-LOX)	0.91	Rabbit reticulocytes	(Hope et al., 1983)
Soybean lipoxygenase	0.45	Soybean	(Whitman et al., 2002)

IC_50_ values established in vitro.

## NDGA Regulates the KEAP1/NRF2 Axis

**Figure 3:**
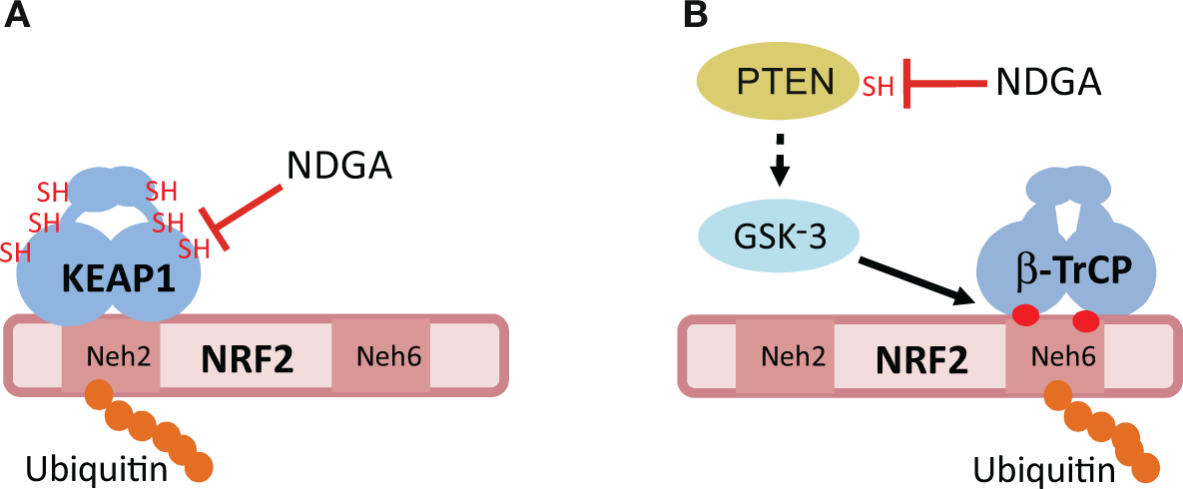
Hypothetical mechanisms of NRF2 activation by NDGA. **(A)** The E3 ligase adapter KEAP1 recognizes the Neh2 domain of NRF2, leading to its ubiquitination and proteasomal degradation. NDGA might inhibit KEAP1 by making adducts with specific cysteines of KEAP1, including Cys151. **(B)** The Neh6 domain of NRF2 is a target for phosphorylation by the Glycogen Synthase Kinase-3 (GSK-3). This phosphorylation creates a site for recognition by the E3 ligase adapter beta-TrCP, leading to its ubiquitination and proteasomal degradation. GSK-3 is inhibited by phosphorylation at its N-terminus by several kinases including AKT. NDGA might inhibit GSK-3 indirectly through adduct formation with the catalytic Cys124 of PTEN. Inhibition of PTEN results in sustained activation of AKT and inhibition of GSK-3, therefore allowing NRF2 to escape this degradation pathway. See text for details of both mechanisms.

A more profound antioxidant action of NDGA than its ROS-scavenging activity is probably related to the activation of the endogenous antioxidant system through the inhibition of the redox sensor KEAP1 (Kelch-like Erythroid Cell–derived Protein with Cap’n’collar Homology (ECH)-associated Protein 1) (**Figure 3A**). KEAP1 is a homodimeric protein that comprises three functional domains: a Broad complex, Tramtrack, Bric-a-brac (BTB) homodimerization domain, an intervening region (IVR) and a C-terminal Kelch domain with a double glycine repeat (DGR) (Canning et al., 2015). KEAP1 is an ubiquitin E3 ligase adapter that binds certain proteins at the Kelch domain and presents them to the E3 ligase complex formed by Cullin 3 and RING-box protein 1 (CUL3/RBX1), leading to their ubiquitination and proteasomal degradation (Zhang and Hannink, 2003; Cullinan et al., 2004; Kobayashi et al., 2004). Therefore, KEAP1 inhibition results in accumulation of these proteins.

The exceptional feature of KEAP1 is that it is a redox sensor. Human KEAP1 contains 27 cysteine residues and several of them can be modified by sulfhydryl reactions under oxidant conditions and by adduct formation with electrophiles, such as NDGA. The most sensitive cysteines for adduct formation are C151, C273, and C288 (Yamamoto et al., 2008). Although experimental evidence is still lacking for NDGA, another catechol, hydroxytyrosol butyrate, appears to interact with the above mentioned cysteines *in vivo* (Funakohi-Tago et al., 2018). However, a single point mutant, C151S, was sufficient to yield KEAP1 refractory to inhibition by the diphenolic compound tert-butylhydroquinone (tBHQ) (Zhang and Hannink, 2003) as well as by carnosic acid, a catechol-type electrophilic compound (Satoh et al., 2008). Therefore, it is tempting to speculate that NDGA inhibits KEAP1 through conversion to its quinone form, followed by adduct formation with C151 of KEAP1 (Satoh et al., 2008).

The best characterized protein interacting with KEAP1 is the transcription factor NRF2, which is considered a master regulator of multiple homeostatic responses (Cuadrado et al., 2018; Cuadrado et al., 2019). NRF2 is a basic region-leucine zipper (bZip) transcription factor that forms heterodimers with the small muscle aponeurotic fibrosarcoma proteins (MAF) K, G, and F (Katsuoka and Yamamoto, 2016). The heterodimer recognizes an enhancer sequence termed Antioxidant Response Element (ARE) that is present in the regulatory regions of over 250 genes (Ma, 2013; Hayes and Dinkova-Kostova, 2014). These genes encode a network of enzymes involved in phase I, II, and III biotransformation reactions, antioxidant metabolism (e.g. generation of NADPH-, glutathione- and thioredoxin-mediated reactions), lipid and iron catabolism, interaction with other transcription factors, as well as autophagy (Hayes and Dinkova-Kostova, 2014; Cuadrado et al., 2019). Although the crystal structure of NRF2 has not yet been reported, its primary sequence reveals several domains termed (Neh)1-6 (NRF2-ECH homology). Under basal homeostatic conditions, the Kelch domains of the KEAP1 homodimer bind one molecule of NRF2 at two N-terminal amino acid sequences in the Neh2 domain: the low affinity binding site (aspartate, leucine, and glycine; DLG) and the high affinity binding site (glutamate, threonine, glycine, and glutamate; ETGE) (Mcmahon et al., 2006; Tong et al., 2006). Thereafter, KEAP1-bound NRF2 is directed to ubiquitination by CUL3/RBX1 and its subsequent degradation by the proteasome. As a result, the constantly synthesized NRF2 is continuously degraded, having a very short half-life of about 20–45 min, depending on the cell type (Mcmahon et al., 2004). However, in an oxidant environment or in the presence of electrophiles (e.g. NDGA), KEAP1 switches towards an inactive form which is no longer capable of promoting NRF2 ubiquitination. As such, NRF2 is stabilized by avoiding proteasomal degradation, and supports cellular adaptation to oxidative stress (Rojo et al., 2012). Definite evidence that NDGA increases NRF2 stability by disrupting the KEAP1/NRF2 interaction was obtained using a chimeric protein that contains the enhanced green fluorescence protein fused to the Neh2 domain of NRF2 (Rojo et al., 2012). The Neh2 tag conferred instability to the chimeric protein and this was prevented in the presence of NDGA. In fact, NDGA promotes the stabilization of the NRF2 protein and upregulation of its gene target *HMOX1* that encodes heme-oxygenase-1 (HO-1), hence conferring cytoprotection against the hydrogen peroxide-induced damage in mouse fibroblasts (Rojo et al., 2012) and against 3-nitropropionic acid in cerebellar granule cells (Guzman-Beltran et al., 2008). Moreover, NDGA induces the nuclear translocation of NRF2 *in vivo* in the rat kidney, leading to the activation of its transcriptional signature and consequent protection against renal oxidative injury and apoptosis in a model of ischemia reperfusion (Zuniga-Toala et al., 2013).

Although for the moment there is no clear experimental evidence, the inhibition of KEAP1 by NDGA might also impact the stability and function of other KEAP1 substrates, besides NRF2. These substrates are still poorly defined but they all share a motif that is identical to or resembles the ETGE motif in the high affinity binding site of NRF2. Empirical evidence for association to KEAP1 has been shown for SQSTM1 (Sequestosome 1), MCM3 (Minichromosome Maintenance Complex Component 3), MCMBP (Minichromosome Maintenance Complex Binding Protein), MCC (Mutated In Colorectal Cancers), the metallopeptidase DPP3 (Dipeptidyl Peptidase 3), SLK (STE20 Like Kinase), MAD2L1 (Mitotic Arrest Deficient 2 Like 1), FAM117B (Family With Sequence Similarity 117 Member B), IKBKB (Inhibitor Of Nuclear Factor Kappa B Kinase Subunit Beta), PGAM5 (PGAM Family Member 5, Mitochondrial Serine/Threonine Protein Phosphatase), and PALB2 (Partner and localizer of BRCA2) (Lo and Hannink, 2006; Goldfarb et al., 2014; Orthwein et al., 2015). However, it should be noted that the disruption of the KEAP1/NRF2 interaction by NDGA might not represent a general mechanism for other KEAP1 substrates and therefore experimental work is needed, not only to establish mechanistic interactions but also to know if these proteins might be functional effectors underlining the anti-tumor and anti-inflammatory activities of NDGA. At least in the case of SQSTM1, there is some indirect evidence indicating that NDGA alters this interaction and inhibits *Mycobacterium tuberculosis* growth in infected macrophages by inducing autophagy (Guzman-Beltran et al., 2016). SQSTM1 is a crucial autophagy protein involved in transporting KEAP1 to autophagosomes. It contains a STGE motif that, upon phosphorylation at the serine residue, resembles the high affinity binding site for KEAP1 (Komatsu et al., 2010). Accordingly, it is possible that, in addition to the induction of autophagy genes through NRF2 activation (Pajares et al., 2016; Pajares et al., 2018), NDGA might modulate autophagy by disrupting the KEAP1/SQSTM1 interaction.

## NDGA Elicits Dual Effects on Various Signaling Cascades

Studies in cell culture indicate that, depending on the context, NDGA may either inhibit or activate the PI3K/AKT axis and the three main MAPK cascades, namely ERK1/2, p38, and JNK. At high concentrations, NDGA disrupts the activation of ERK and AKT signaling pathways activated by IGF-I (insulin-like growth factor-1) and induces apoptosis (Meyer et al., 2007), the effect being highly important for cancer treatment but also for unwanted side-effects. At least in the case of the PI3K/AKT pathway, the NDGA-mediated inhibition resembles the effect of high concentrations of hydrogen peroxide, which lead to elevated levels of intracellular ROS and ceramide. These intermediary molecules trigger the down-regulation of AKT by dephosphorylation and subsequent proteolysis (Martin et al., 2002). However, low micromolar concentrations of NDGA were found in most reports to activate the mentioned kinase pathways. This effect is most likely due to the redox cycling nature of NDGA which, depending on dosing and cell type, induces a mild oxidative stress and alteration of redox sensitive cysteines in particular proteins by sulfhydryl modification, as well as GSH depletion. For instance, NDGA-mediated apoptosis in the murine prolymphocytic cell line FL5.12 was shown to be independent of LOX inhibition but was partially related to p38 activation, and was prevented by the antioxidant N-acetyl cysteine (Deshpande and Kehrer, 2006). In fact, redox cycling polyphenols like NDGA can alter the balance between phosphorylation and dephosphorylation by inhibiting redox-sensitive phosphatases (Ostman et al., 2011). An example is PTEN (Phosphatase and Tensin Homolog) in which a catalytic cysteine is required to form a covalent intermediate with the phosphate group in order to be eliminated from the substrate protein. Evidence gathered with the diphenolic compound tBHQ indicates that this cysteine is susceptible to form adducts with the oxidized benzoquinone form of tBHQ, hence triggering PTEN inhibition and consequent AKT activation (Ostman et al., 2011; Rojo et al., 2014). Assuming that the catechol quinone derived from NDGA oxidation behaves as this benzoquinone, it is most likely that NDGA is activating the PI3K/AKT pathway by inhibiting PTEN.

The modulation of MAPKs by NDGA is less clear. It is noteworthy that dual specificity phosphatases (DUSPs), which are involved in shutting down these pathways, exhibit a catalytic cysteine, similar to PTEN. Therefore, a comparable inhibitory effect of NDGA as described for PTEN is suggested, resulting in MAPK activation (Rios et al., 2014). NDGA seems to use this mechanism also in the case of JNK for protection against cerebral ischemia/reperfusion (Liu et al., 2012). The JNK upstream kinase ASK1 is sensitive to oxidative stress through interaction with thioredoxin, and it was demonstrated that the electrophile acrolein alkylates thioredoxin reductase-1 and thioredoxin-activating JNK (Randall et al., 2013). More experimental work is required to determine if NDGA inhibits catalytic cysteines, in particular phosphatases and other proteins, and impacts the signaling networks that are critically involved in various pathologies.

## NDGA Can Activate NRF2 in a KEAP1-Independent Manner

As described above, NDGA inhibits the redox sensor KEAP1, leading to NRF2 activation. However, the fact that NDGA can stabilize the protein levels of NRF2, even in fibroblasts derived from *Keap1*-knockout mouse embryos, points towards other mechanisms besides KEAP1 inhibition for regulating NRF2 stability. It was found that NDGA stabilizes NRF2 in a KEAP1-independent manner through phosphorylation of its Neh6 domain (Rojo et al., 2012). In fact, the Neh6 domain was shown to confer instability to a chimera made of cyan fluorescent protein and the Neh6 domain, and this effect was prevented by NDGA. In this domain, NRF2 presents two motifs, DSGIS and DSAPGS, that, upon phosphorylation, are converted into a degradation signal recognized by the E3 ligase adapter beta-TrCP (beta-transducin repeat-containing protein) (Cuadrado, 2015). This protein connects phospho-NRF2 with the CUL1-RBX1 ubiquitin ligase complex, and promotes its degradation (**Figure 3B**). The kinase involved in phosphorylation of the DSAPGS motif was not identified (Chowdhry et al., 2013), but it is known that the DSGIS motif is phosphorylated by the serine/threonine protein kinases GSK-3α and GSK-3β (Cuadrado, 2015). GSK-3 is an active kinase under resting conditions, but it is inhibited upon growth factor signaling by phosphorylation of its N-terminal domain (Serine 21 and Serine 9 in GSK-3α and GSK3β, respectively) (Rada et al., 2011; Rada et al., 2012). A very well established kinase involved in GSK-3 phosphorylation is AKT (Van Weeren et al., 1998; Woodgett, 2005). NDGA mimics growth factor signaling to activate AKT, rendering GSK-3 inactive and preventing the formation of the DSGIS phosphodegron (Rada et al., 2011; Rada et al., 2012). It has also been reported that p38 inhibits human GSK-3β through phosphorylation of T390 (Thornton et al., 2008) and therefore NDGA might also contribute to inhibition of GSK-3 and subsequent stabilization of NRF2 *via* activation of the p38 pathway, but this mechanism needs remains to be demonstrated.

## NDGA as a Therapeutic Agent

### NDGA Protects Against Renal Damage

Deterioration of renal function is associated with impairment of the electrolyte and acid balance, resulting in irreversible kidney damage and renal necrosis. Therapy may include hemo- and peritoneal dialysis, and a kidney transplant is required in severe cases. All these therapeutic strategies are helping in alleviating symptoms, but cannot prevent or reverse renal damage. In this context, NDGA has emerged as a novel promising candidate considering that oxidative stress and inflammation are the major pathological mechanisms of nephrotoxicity.

Firstly, the antioxidant capacity of NDGA has been evaluated in renal dysfunction induced by ferric-nitrilotriacetate. NDGA prevented the reduction in the expression of key antioxidant enzymes, such as glutathione-S-transferase, glutathione-reductase, glucose-6-phosphate dehydrogenase, and catalase, that are induced by ferric-nitrilotriacetate. Accordingly, NDGA increased GSH concentration and reduced the levels of oxidative damage markers such as lipid peroxides and oxygen peroxide (Ansar et al., 1999). Similar results were obtained in a later study of streptozotocin-induced nephropathy in rats. Administration of NDGA prevented the increase in renal malondialdehyde levels and the decrease in the renal GSH content, superoxide dismutase and catalase activities, paralleled by the decrease of proteinuria (Anjaneyulu and Chopra, 2004). Moreover, the effect of NDGA on K_2_Cr_2_O_7_-induced nephrotoxicity and the associated oxidative/nitrosative stress indicates that when this drug is administered in mini osmotic pumps, it can reduce the levels of the oxidative and nitrosative stress markers 4-hydroxy-2-nonenal and 3-nitrotyrosine, respectively (Yam-Canul et al., 2008). Histologic analysis of slices from K_2_Cr_2_O_7_-treated rats showed extensive tubular damage, and most of cortical tubules exhibited epithelial atrophy and casts. Interestingly, K_2_Cr_2_O_7_/NDGA-treated rats had lesser tissue damage and fewer epithelial tubular cells were affected. In addition, the levels of urinary N-acetyl-β-d-glucosaminidase, serum creatinine and serum glutathione peroxidase activity were actually normalized after NDGA treatment (Yam-Canul et al., 2008). These results are further supported in rodent models of the human idiopathic nephrotic syndrome, which is based on puromycin aminonucleoside-induced nephrosis. Ultrastructural investigations by electron microscopy showed that podocyte morphology was changed after induction of nephrosis but recovered after NDGA administration. Moreover, protein excretion in urine was significantly lower in the animal groups treated with NDGA than in the control groups (Lee et al., 2009). In diabetic nephropathy, NDGA was also shown to improve renal function by decreasing the ratio of urinary albumin to creatinine, paralleled by a decrease in serum lipid peroxide levels (Gad, 2012). Moreover, NDGA was shown to accelerate the recovery of the renal function after cisplatin treatment. In line with the results of previous studies, NDGA pretreatment prevented oxidative and nitrosative stress, as well as inflammation (restoration of the levels of the anti-inflammatory IL-10 cytokine in the kidney), and preserved the renal function (Mundhe et al., 2019). In a model of polycystic kidney disease, which is characterized by increased levels of renal cyclooxygenase (COX)-derived eicosanoids, NDGA decreased the levels of prostaglandin PGF_2_ and LOX-derived metabolites, but this inhibition was not clearly associated with changes in the renal function or disease progression (Ibrahim et al., 2015). In conclusion, NDGA exerts renal protective actions in preclinical models, hence suggesting its therapeutic potential for the treatment of patients with kidney failure and other associated complications.

### NDGA Protects Against Liver Disease

The role of NDGA in liver protection has been widely addressed experimentally using various models that mimic key hallmarks of acute hepatotoxicity. Diverse studies have pointed out that both metabolic syndrome and liver damage induced by an unbalanced diet, were improved when NDGA was co-administered. Obesity, insulin resistance, hepatic steatosis and liver fibrosis were attenuated by NDGA in mice fed with high trans-fat, cholesterol, and fructose diet (Han et al., 2019). In mice submitted to a typical American “fast food” diet, NDGA normalized insulin sensitivity, but not glucose intolerance, body and fat pad weight, ALT, AST, and liver triglycerides (Chan et al., 2018). By contrast, in mice fed with a high-fat diet for 8 weeks, NDGA reduced weight gain, fat pad mass and hepatic triglyceride accumulation, and improved serum lipid parameters (Zhang et al., 2016). Further analysis showed that this NDGA effect is underlined by the activation of the AMP-activated protein kinase (AMK) in the liver and in HepG2 hepatocytes. Specific analysis of the mechanism through which NDGA exerts its anti-hypertriglyceridemic action was determined in response to a high-fructose diet. Oral administration of NDGA decreased the plasma levels of glucose, insulin, triglycerides and fatty acids, increased hepatic mitochondrial fatty acid oxidation and attenuated hepatic accumulation of triglycerides (Zhang et al., 2016). In addition, chronic administration of NDGA to obese mice (ob/ob) significantly improved plasma triglycerides levels, inflammatory chemokines levels, hyperinsulinemia, insulin sensitivity and glucose intolerance, while enhancing the rate of fatty acid oxidation (Zhang et al., 2013). Type 2 diabetes mellitus is a complex disease with alterations in metabolic and inflammatory markers which could be spontaneously developed by Stillman Salgado rats. Dain et al. (2016) analyzed in these rats the effects of ω-3 polyunsaturated fatty acids supplementation with or without NDGA added, and they observed that NDGA treatment ameliorated inflammatory, metabolic, and oxidative stress markers (Dain et al., 2016).

NDGA deeply impacts the transcriptomic profile of the liver. Global transcriptional changes have been analyzed in response to chronic administration of NDGA in the context of a high-fructose diet. NDGA upregulated the expression of several genes involved in fatty acid oxidation (*ACOX1*, *CPT1B*, *CPT2*, *ACADVL*, *ECI1,* and *EHHADH*) and *PPARα*, which is the transcription factor considered as the master regulator of fatty acid oxidation. On the other hand, the expression of some lipogenic genes and relevant transcriptional factors were reduced in the NDGA-treated animals (*GCKR, GCK, ACLY, FASN, SCD1, ELOVL2, ELOVL5, FADS1 FADS2, DGAT2, ARF3, HMGCR, INSIG1, INSIG2*). NDGA differentially affected the genes encoding fatty acid transporters, acetyl CoA synthetases, elongases, fatty acid desaturases, and lipid clearance proteins (Zhang et al., 2015). Some of these findings were validated by qRT-PCR and immunoblot in independent studies. NDGA downregulated the protein level of SREBP-1 and therefore of its target genes, *ACC* and *FAS*. In turn, it upregulated the levels of proteins involved in fatty acid oxidation, such as PPARα, PGC-1, CPT-1L, UCP2 and UCP3 (Lee et al., 2010). The transcription factor PPARα plays a crucial role in the response to NDGA. Both the mRNA and nuclear protein levels of PPARα were upregulated by NDGA (Lee et al., 2010; Zhang et al., 2013). In fact, NDGA increased *PPARα* promoter activity in AML12 hepatocytes. Very relevant, reduction of PPARα expression by siRNA abrogated its stimulatory effect on fatty acid catabolism. Likewise, no stimulatory effect of NDGA on hepatic fatty acid oxidation was observed in the liver of PPARα-deficient mice (Zhang et al., 2013). These findings suggest that NDGA ameliorates hypertriglyceridemia and steatosis primarily by altering the expression of genes encoding key enzymes and transcription factors involved in *de novo* lipogenesis and fatty acid oxidation.

Altogether, these studies strongly suggest that the antioxidant and anti-inflammatory properties of NDGA are involved in its kidney protective effect; whereas its role in liver is closely related to its capacity to increase lipid catabolism. However, we might be aware that NDGA concentration is a key issue to correctly interpret the experimental data. For instance, NDGA exhibits adverse pro-oxidant effects on clone-9 rat hepatocyte cultures in the concentration range of 20-100 μM, while it has beneficial antioxidant effects on rat alveolar macrophages and Chinese hamster lung fibroblasts at concentrations below 10 μM (Robison et al., 1990). As it will be discussed in section *Adverse Effects of NDGA*, a growing body of evidence supports the fact NDGA is deeply impacting kidney and liver physiology, precluding is clinical development.

### NDGA Protects Against Neurodegeneration

Extensive data from animal models and human samples provide strong evidence for an early role of redox and neurotransmitter imbalance, inflammation, mitochondrial dysfunction, and altered proteostasis as common mechanisms in the pathogenesis of neurodegenerative diseases that are clinically characterized by progressive loss of neurons and compromised motor or cognitive functions (Ibanez et al., 2004; Alzheimer’sassociation, 2016). Existing therapeutic approaches do not control the unrelenting progression of neurodegeneration, and the therapeutic approaches designed to target individual signaling pathways have failed in clinical studies. As we have reviewed here, NDGA exerts protective effects against various deleterious signals involved in neurodegeneration.

#### NDGA Modulates Oxidative Stress in the Brain

Compared with other organs, the brain consumes very high oxygen amounts, has low antioxidant defense mechanisms and a high content of polyunsaturated fatty acids that are readily prone to be oxidized. Mitochondrial impairment, resulting in ROS overproduction, is also an underlying mechanism of neurodegeneration (Cenini et al., 2019). These features make the brain especially vulnerable to oxidative stress-induced damage. Therefore, the use of antioxidant compounds which are able to restore the redox balance may greatly help to keep under control the susceptibility of the brain to oxidative damage.

The intrinsic antioxidant activity of NDGA was evidenced in rat brain homogenates by measuring the production of thiobarbituric acid reactive substances, formation of peroxy-lipids and carbonyl-proteins. These redox markers were significantly reduced when the brain extracts were incubated with NDGA (Shishido et al., 2001; Czapski et al., 2012). The neuroprotective effect of NDGA has been also evaluated in neuronal cultures submitted to generic oxidant compounds such as oxygen peroxide (Guzman-Beltran et al., 2008) or iodoacetate (Cardenas-Rodriguez et al., 2009) used as inhibitor of glyceraldehyde-3-phosphate dehydrogenase. In both experimental settings, the neuroprotective effects exerted *in vitro* by NDGA were associated with the prevention of oxidative stress. Additionally, NDGA prevented the increase in ROS and calcium levels, as well as neuronal injury in an *in vitro* model of Alzheimer’s disease (AD), consisting in the treatment of neuronal cultures with amyloid beta (Aβ) (Goodman et al., 1994). Moreover, Guzman-Beltran et al. (2008) demonstrated that NDGA protects neurons against 3-nitropropionic acid, a chemical model of Huntington Disease (HD), through the activation of the NRF2 transcription factor. Furthermore, this study pointed out the stimulatory activity of NDGA on the NRF2 target *HMOX1*, which has an important role in neuroprotection (Guzman-Beltran et al., 2008).

Further demonstration of the antioxidant activity of NDGA in the brain has been done using *in vivo* models characterized by increased oxidative stress. For instance, diabetic encephalopathy is a chronic complication of diabetes mellitus that affects the central nervous system. Plasma and brain samples of diabetic rats exhibited higher levels of oxidative stress markers, gamma-glutamyltranspeptidase activity, and hydro-/lipoperoxides than those found in control rats. Interestingly, the levels of those markers were reduced when the rats were injected monthly with NDGA for 12 months (Diaz-Gerevini et al., 2019). In the striatal neurons of the R6/2 mouse model of HD, NDGA markedly reduced the levels of 4-HNE (marker of lipid peroxidation) and preserved mitochondrial morphology and ATP generation. These beneficial effects of NDGA were associated with an increase of the lifespan of HD mice (Lee et al., 2011).

#### NDGA Regulates Neurotransmission

Alterations in the cholinergic neurotransmission at the cortex and hippocampus are important hallmarks in many forms of dementia (Wilcock et al., 1982; Muir, 1997). In fact, acetylcholinesterase (AChE) inhibitors are currently the main therapeutic tool for restoring acetylcholine levels in the pathogenesis of AD (Arvanitakis et al., 2019). Virtual screening of diverse natural products against AChE revealed that NDGA was among the top scored compounds with an IC_50_ value of 46.2 μM. Moreover, structural modifications of NDGA were performed *in silico* to obtain derivatives with improved blood brain barrier penetration and improved activity in the central nervous system. The new NDGA derivatives were more lipophilic, less flexible and had lower molecular weight than NDGA. AchE binding analysis showed higher binding affinity for the designed ligands, probably due to higher hydrogen bonding and π–π interactions (Remya et al., 2013).

Chronic excitotoxicity plays a role in many neurodegenerative diseases, having a particular relevance in amyotrophic lateral sclerosis (ALS). Excitotoxicity results from excessive activation of glutamate receptors, and leads to loss of neuronal structures including dendrites and cell bodies (Meldrum and Garthwaite, 1990; Limanaqi et al., 2019). Considering that synaptic accumulation of glutamate is detrimental to neurons, drugs like NDGA, that are capable of increasing glutamate uptake by the astrocytes, might be therapeutically beneficial (Lynch et al., 1989). Indeed, subcutaneous administration of NDGA for 30 days in mice, increased glutamate uptake in synaptosomes from the spinal cord (Boston-Howes et al., 2008). In turn, the effect of NDGA in a mouse model of ALS (SOD1-G93A mouse) is slightly controversial. Initially, oral NDGA administration significantly extended lifespan by 10%, slowed motor dysfunction and triggered a reduction in gliosis and neuron damage (West et al., 2004). However, a later study did not find that NDGA could extend life span of these mice when administered subcutaneously (Boston-Howes et al., 2008).

#### NDGA Limits Neuroinflammation

Chronic inflammation plays a critical role in neurodegenerative disease and therefore immunosuppressive/modulatory strategies hold great promise. For instance, immune interventions have been successfully applied in the clinic to treat multiple sclerosis (Rieckmann et al., 2008). Several studies analyzed the anti-inflammatory role of NDGA in the context of LOX inhibition and the resulting reduction of harmful arachidonic acid (AA)-derived metabolites. NDGA was shown to prevent ischemic/reperfusion damage in a model of cultured rat cortical neurons that were subjected to oxygen-glucose deprivation (OGD) (Liu et al., 2012). In this study, NDGA reduced the levels of phospho-JNK and phospho-c-JUN, preventing neuronal apoptosis through 12/15-LOX inhibition. In addition, NDGA protected neurons in stroke models based on permanent or transient occlusion of the middle cerebral artery followed by reperfusion (Liu et al., 2012). Moreover, NDGA significantly attenuated post-ischemic learning and memory impairment after transient four-vessel occlusion in rats. Furthermore, consecutive administration of NDGA for 4 days significantly reduced the post-ischemic neuronal death of pyramidal cells in the rat hippocampus (Shishido et al., 2001). In a Parkinson’s disease model, the toxic effect of nitric oxide (NO) on GSH-depleted primary midbrain cultures was partially prevented by NDGA (Canals et al., 2003). The anti-inflammatory effects of NDGA were also evaluated in a spinal cord injury model which is characterized by inflammation. In this context, NDGA significantly decreased myeloperoxidase (MPO) levels, as an indicator of neutrophil activity, and also the number of macrophages/microglia cells. In addition, NDGA suppressed the expression of the pro-inflammatory cytokines IL-1β and TNF-α. Of utmost importance, histological analysis of the spinal cord showed an increased number of neurons after NDGA administration and the extent of secondary damage, measured as the number of apoptotic cells and proliferating astrocytes, was significantly decreased (Xue et al., 2013).

Modulation of the IFNγ response by NDGA deserves special attention. It has been proposed that in rat astrocytes, NDGA suppresses the pro-inflammatory response mediated by IFNγ in a LOX-independent manner (Jeon et al., 2005). Thus, in the presence of NDGA, the expression of pro-inflammatory factors such as IRF-1 (interferon regulatory factor-1), MCP-1 (monocyte chemotactic protein-1), interferon-gamma inducible protein-10 (IP-10), and the CXCL10 chemokine) were significantly reduced, as well as the levels of phospho-JAK and phospho-STAT. However, the 5-LOX products LTB4 and LTC4 were not detected in cells treated with IFNγ. In addition, two other 5-LOX inhibitors (Rev5901 and AA861) did not mimic the effect of NDGA, and addition of 5-LOX metabolites did not reverse the NDGA-driven suppression of STAT. These results suggest that NDGA regulates IFNγ-mediated inflammation through mechanisms that are not related to LOX inhibition and might be the result of combined mechanisms, possibly related to NRF2 activation (Cuadrado et al., 2018).

#### NDGA Prevents Proteinopathy

Proteinopathy is a pathological condition characterized by the formation of protein deposits in the form of amyloid fibrils. In the brain, protein aggregates encompass dimers, oligomers, protofilaments, and fibrils (Stefani, 2010). Thus, misfolded aggregates of α-synuclein are found in PD, β-amyloid (Aβ) plaques and hyper-phosphorylated TAU neurofibrillary tangles in AD, huntingtin in HD, superoxide dismutase 1, and TAR DNA binding protein 43 (TDP-43) in ALS, etc. A growing body of evidence supports a connection between NDGA and amyloidosis. Nusrat et al. (2016) studied this concept using egg white lysozyme (HEWL) as a model protein for amyloidosis. NDGA interferes with the amyloid fibrillogenesis process by hydrophobic interaction with the amino acid residues found in the highly prone amyloid fibril forming region of HEWL, as demonstrated by molecular docking results (Nusrat et al., 2016). Previous studies have also addressed the role of NDGA in Aβ or α-synuclein oligomerization. Particularly, by analyzing the fluorescence derived from the Aβ probe, thioflavin T, Yamada’s group showed that NDGA inhibits Aβ fibril formation (Naiki et al., 1998; Ono et al., 2004) and disaggregates Aβ fibrils formed *in vitro* (Ono et al., 2002). These results were confirmed and extended to Aβ protofibrils in a later study, where the authors have combined fluorescence analysis of the thioflavin T probe with electron microscopy. However, the authors established that the NDGA-induced decrease in thioflavin T fluorescence was not accompanied by a reduction in Aβ aggregate size or quantity. To elucidate these controversial results, NDGA supplementation was given to AD transgenic mice (Tg2576) for 10 months starting at the age of 5 months. It was found that Aβ deposition, assessed immunohistochemically, was significantly decreased in the brain of NDGA-treated mice (Moss et al., 2004).

NDGA inhibits dose‐dependently α-synuclein oligomerization (Takahashi et al., 2015) due to the binding of multiple molecules of NDGA per α-synuclein molecule (Haney et al., 2017). Recently, it was shown that NDGA induced modest but progressive compaction of monomeric α-synuclein, hence preventing its aggregation into amyloid-like fibrils. This conformational remodeling preserved the dynamic adoption of α-helical conformations that are essential for physiologic membrane interactions (Daniels et al., 2019). The modulation of α-synuclein dynamics by NDGA was studied in connection with climbing ability in a *Drosophila* PD-model expressing normal human α-synuclein in neurons. Diet supplementation with NDGA for 24 days improved in a dose-dependent manner the locomotor dysfunction exhibited by the mutant flies (Siddique et al., 2012).

### NDGA Has Anti-Cancer Action

NDGA exerts *in vitro* anti-cancer effects on various types of tumor and leukemia cell lines in the concentration range 1–100 µM. The mechanisms underlining the observed effects might differ depending on NDGA concentration and the type of cancer cells. In tumor-bearing animal models NDGA was tested in the dose range of 0.750–100 mg/kg body weight (Hernandez-Damian et al., 2014). As shown in **Table 3**, NDGA holds great promise as a therapeutic agent for several types of cancer, as extensively demonstrated by preclinical studies on tumor cell lines and animal/human tumors. The rationale behind the anti-tumor action resides in the fact that most cancer cells are characterized by low-grade oxidative stress and inflammation that provide a survival and growth advantage in the hostile tumor microenvironment, as well as resistance to therapy (Manda et al., 2015). **Table 4** summarizes the nine clinical studies on NDGA and its analog terameprocol (see section *NDGA-Analogs as Novel Therapeutic Small Molecules*), but only two of them have reported results. They will be discussed in the following subsections.

**Table 3:** Some preclinical studies on the effect of NDGA and NDGA analogs in cancer.

Cells/Animal models	Compound	Concentration	Effect/Mechanism of action	Reference
Breast cancer cells: trastuzumab-naive and trastuzumab-refractory HER2-overexpressing SK-BR-3 and BT-474 human cells	NDGA	25-100 µM	Induces DNA fragmentation, cleavage of poly(ADP-ribose) polymerase and caspase-3, and promotes cell death of both trastuzumab-naive and trastuzumab-refractory HER2-overexpressing breast cancer cells. NDGA and trastuzumab suppressed proliferation and survival of trastuzumab-refractory cells to a greater degree than either agent alone	(Rowe et al., 2008)
SiHa cervical cancer cells (grade II squamous cell carcinoma)	NDGA	20-100 µM	Growth inhibition induced by up-regulating p21	(Gao et al., 2011)
Human SW 850 pancreatic and C4-I cervical cancer cells	NDGA	25 µM	Inhibits anchorage-independent growth of pancreatic and cervical tumor cells. Increases apoptosis (cells exhibiting fragmented DNA at 12 h post-exposure to NDGA). Disrupts the actin cytoskeleton and activates JNK and p38^mapk^ before cell detachment	(Seufferlein et al., 2002)
Athymic NMRI/nu-nu mice transplanted with human SW 850 pancreatic and C4-I cervical cancer cells		90 μM	Moderately inhibits tumor growth *in vivo* (delays the growth of pancreatic and cervical human tumors in athymic mice)	
PC3 human prostate cancer cells	NDGA	20-50 µM	Inhibits cell growth in a concentration-dependent and increases intracellular calcium levels (EC_50_ = 30 µM)	(Huang et al., 2004)
	NDGA	10 and 20 µM	Inhibits cell migration and tumor metastasis. Suppresses neuropilin 1 (NRP1) function by downregulating its expression, leading to attenuated cell motility, cell adhesion to extracellular matrix, and FAK signaling in cancer cells	(Moody et al., 1998)
PC3 xenografts (14 and 28 days treatment)	NDGA	50 and 100 mg/kg	Blocks the expression and consequently the function of NRP1 in tumor xenografts	
NCI-H1264 lung cancer cells	NDGA	3–10 µM	Decrease tumor cell growth (3µM) and colony number (10µM)	(Moody et al., 1998)
Non-small-cell lung cancer xenografts (NCI-H157 or H1264 cells) in athymic BALB/c nude mice		0.1% in drinking water for 4 months	Inhibits lung cancer growth and prevents lung carcinogenesis	
Chemically induced (urethane) adenoma in A/J mice				
TA3 grown in CAF 1 Jax mice and 786A cells grown in A Swiss mice	NDGA	25–100 µM (TA3 cells) 42–126 µM (786A cells)	Inhibits the respiration rate of tumor cell lines by preventing electron flow through the respiratory chain, hence decreasing ATP levels, cell viability and culture growth rates.	(Pavani et al., 1994)
Human leukemic HL‐60 and U‐937 cell lines	NDGA	3–60 µM	Decreases cell viability in a dose-dependent manner IC_50_ at 48 h in HL‐60: 5.8 ± 0.5 IC_50_ at 72 h in U-937 cells: 7.5 ± 1.0. Inhibits glucose uptake leukemic cell lines through a non-competitive mechanism.	(Leon et al., 2016)
Human red blood cells		0.1–100 µM	Blocks hexose transport in human red blood cells and displaces pre-bound cytochalasin B from erythrocyte ghosts (*K*_Dapp_=4.5 μm), possibly through a direct interaction with the glucose transporter GLUT1.	
Lymphatic leukemia P388 cells, grown in the abdominal cavity of DBA2 mice	NDGA	0.01–30 µg/ml	Induces apoptotic death (IC_50__=_ 0.66 μg/ml).	(Bibikova et al., 2017)
Multiple myeloma cells (RPMI-8226, LP-1, KMS-18 and KMS-11)	NDGA	0.1–40 µM	Inhibits FGFR3 autophosphorylation both *in vitro* (dramatic reduction induced by 0.5 μM NDGA) and *in vivo (IC*_*50*__=_ *10 µM).* Decreases MAPK activation which results in increased apoptosis.	(Meyer et al., 2008)
Acute lymphoblastic leukemia (ALL) (MOLT-4, Jurkat-FADD deficient)	NDGA	2 µM	Protects ALL cells from lipid peroxidation, ROS generation and cell death induced by the small molecule RSL3 (inducer of ferroptosis).	(Probst et al., 2017)
Proliferating C3, C33a, CEM-T4, and TC-1 cells	Terame procol	10–100 µM	Arrests proliferation at the G2 phase (10–40 µM). Reduces mRNA levels and protein production of the cyclin-dependent kinase CDC2 (40 µM), resulting in the inactivation of the maturation promoting factor CDC2/cyclin B complex.	(Heller et al., 2001)
C3-cell induced C57bl/6 mouse tumor model	Terame procol	20 mg/day intratumoral	Substantial tumoricidal activity that correlated with a reduction in tumor cell CDC2 protein levels.	
Leukemic cell lines (OCI-AML3, U937, U937neo, U937XIAP, Jurkat, JurkatI2.1, HL-60, HL-60neo, HL-60Bcl-2, and HL60Bcl-XL c, KBM5 cells), and acute myeloid leukemia (AML) blasts	Terame procol	5–40 µM	Inhibits growth and induces cell death in leukemic cell lines and blasts from AML patients. Significant inhibition of AKT phosphorylation was observed in M4N treated OCI-AML3 cells. The effects are not mediated by a mechanism not mediated by Cdc2 and survivin inhibition or by the extrinsic and the mitochondrial apoptotic pathways.	(Mak et al., 2007)
Nude mice with xenografts of hepatocellular (Hep 3B) prostate (LNCaP) colorectal (HT-29) breast (MCF7) carcinomas; erythroleukemia (K-562)	Terame procol	2 mg/day for 3 weeks (i.p.) 300 mg/day for 3 weeks (oral)	Suppresses the *in vivo* growth of xenografts. Induces growth arrest and apoptosis in both xenograft tumors and in tumor cells grown in culture, accompanied by reduction in both Cdc2 and tumor-specific s37urvivin gene expression.	(Park et al., 2005)
ICR mice		44 mg/kg (5-40 min, 2–16 h)	Absolute bioavailability of oral M4N: approximately 88%. Minimal drug-related toxicity.	
Glioma stem-like cells (GSLC)	*dl*-NDGA (Nordy)	5–60 µM	Inhibits self-renewal and induces differentiation of tumor stem cells *in vitro* (10 μM) and *in vivo.* Inhibits 5-LOX (19 µM). Reduces the GSLC pool through a decrease in the CD133+ population and abrogates clonogenicity. This occurs apparently *via* astrocytic differentiation, by up-regulating GFAP and down-regulating stemness related genes, rather than by inducing apoptosis of GSLCs.	(Wang et al., 2011)
Xenografted glioma		13.5 mg/kg or 27 mg/kg every other day (8 times)		

**Table 4:** Clinical trials with NDAG or its derivate terameprocol.

Compound	Identifier	Disease	Phase	Status	Results
NDGA	NCT00678015	hormone-sensitive non-metastatic prostate cancer	phase 2	terminated	(Friedlander et al., 2012)
NDGA	NCT00313534	Nonmetastatic Relapsed Prostate Cancer	phase 1	terminated	No
terameprocol	NCT00404248	Recurrent High-Grade Glioma	phase 1	completed	(Grossman et al., 2012)
terameprocol	NCT00154089	Cervical Intraepithelial Neoplasia	phase 1/2	completed	No
terameprocol	NCT00259818	Recurrent or Refractory Solid Tumors	phase 1	completed	No
terameprocol	NCT00057512	Refractory Malignant Tumors of the Head and Neck	phase 1	completed	No
terameprocol	NCT00664677	Leukemia	phase 1	terminated	No
terameprocol	NCT00664586	Refractory Solid Tumors	phase 1	terminated	No
terameprocol	NCT02575794	Recurrent High Grade Glioma	phase 1	active	No

See text for discussion of outcomes of the two studies that reported results.

#### NDGA Exerts Anti-Cancer Effects by LOX Inhibition

Several types of cancer cells exhibit altered LOX expression or activity, and this is highly differentiated according to the involved LOX isoform, cancer cell type and the context. Moreover, the interplay between tumor cells and stroma (epithelial, endothelial and immune cells) is critically involved in tumor progression from the LOX perspective. As demonstrated in colorectal cancer (Mariani et al., 2014), inflammation and necrosis within the tumor niche lead to the recruitment of monocytes and their polarization towards a pro-inflammatory phenotype, hence reinforcing inflammation in the tumor microenvironment through increased production of pro-inflammatory cytokines (TNFα, IL-12 and IL-23). Moreover, stromal, epithelial and endothelial cells express LOXs (5-LOX, 12-LOX), and COX2 which generate potent inflammatory mediators (leukotrienes and prostaglandins) that trigger the recruitment of neutrophils, and consequently amplify inflammation through increased production of ROS and matrix metalloproteinases (MMP). If the inflammatory stimulus is switched-off, the stromal and epithelial cells expressing 15-LOX produce pro-resolving lipoxins, which block neutrophils migration, stimulate the phagocytosis of apoptotic cells by macrophages and polarize macrophages to an anti-inflammatory phenotype. If the stimulus is not resolved, stromal and epithelial cells amplify the inflammatory signals in the tumor niche (IL-1, IL-8, 5-LOX, and 12-LOX), hence inhibiting neutrophils’ apoptosis and sustaining tumor growth and metastasis through increased production of ROS and MMPs.

The LOX status plays a critical role in several types of cancer. Increased levels of 12-HETE, the arachidonic acid metabolite derived from 12-LOX activity, promotes the proliferation of human colon, pancreatic and breast cancer cell lines, and plays an important role in cell adhesion and metastasis (Yang et al., 2012). AA turnover was found to be 10 times higher in prostate tumors than in the corresponding normal tissue, and elevated mRNA expression of 12-LOX was found more frequently in advanced stage, high-grade prostate cancer (Gao et al., 1995). 12-LOX sustains the proliferation of prostate cancer cells, favors their metastasis to the bone and stimulates angiogenesis (Tang and Honn, 1999). 12-HETE and 5-HETE, the products of 12-LOX and 5-LOX respectively, were shown to act as pro-growth and pro-survival factors for human prostate cancer cells by inducing a tumor-sustaining inflammatory and oxidative microenvironment. Additionally, 5-LOX promotes the growth of prostate tumor cells by over-activating the c-Myc oncogene, as demonstrated by a whole genome gene expression study (Sarveswaran et al., 2015). Furthermore, 5-LOX and 12-LOX appear to be promising biomarkers and therapeutic targets for prostate cancer stem cells (Yin et al., 2011).

In turn, low levels of the 15-LOX-2 isoform and consequently decreased 15-HETE formation were found as distinctive alterations of AA metabolism in prostate cancer cells. 15-LOX enzymes may exert anti-tumoral effects in particular types of tumors (Klil-Drori and Ariel, 2013) by promoting apoptosis, ferroptosis, or autophagy (Li et al., 2018). For instance, 15-LOX-1 contributes to inflammation resolution through its 13-HODE product (13-hydroxyoctadecadienoic acid) derived from linoleic acid, and has an important role in the terminal differentiation of normal cells. 15-LOX-1 is down-regulated in human colorectal polyps and cancers (Il Lee et al., 2011). In turn, the 5-LOX isoform contributes to tumorigenesis in colorectal cancer, mostly due to the infiltration of mast cells (Cheon et al., 2012; Mashima and Okuyama, 2015).

Altogether, these data highlight that 12- and 5-LOX inhibitors with antioxidant properties, like NDGA, could be efficiently used for targeting simultaneously critical pathological mechanisms in cancer such as proliferation, defective apoptosis, metastasis and angiogenesis, as well as the chronically enhanced oxidative stress in the tumor niche.

#### Anti-Cancer Effects of NDGA Are Mediated Also by Tyrosine Kinases

NDGA decreases tumor progression in various preclinical models by inhibiting metabolic enzymes that are critically involved in prostate, lung, esophageal and skin cancers (e.g. fatty acid synthase and LOX enzymes) (Lu et al., 2010). Additionally, NDGA inhibits tumor-relevant receptor tyrosine kinases and downstream signaling related to the IGF-1 receptor and the downstream protein serine/threonine kinase AKT, along with the c-ErbB2/HER2/Neu receptor in breast cancer cells and in tumor-bearing mice (Youngren et al., 2005; Li et al., 2009; Lu et al., 2010). Of utmost importance for cancer treatment, NDGA inhibits signaling pathways mediated by the transforming growth factor β (TGF-β) type I receptor which triggers Smad2 translocation to the nucleus and its subsequent phosphorylation (Li et al., 2009). As demonstrated in pancreatic cancer cells, TGF-β functions as a tumor suppressor in the early stage of neoplasia, but acts as a pro-tumoral stimulus at later stages (Truty and Urrutia, 2007). Therefore, the moment of NDGA administration during tumor progression may be critical for efficiently controlling tumor growth.

#### NDGA Promotes Death of Cancer Cells

Another mechanism through which NDGA seems to exert anti-tumoral effects is by directly promoting the death of various tumor cells or by sensitizing tumor cells to other anti-tumor agents. For instance, NDGA increases the susceptibility of prostate and colorectal tumor cells to TRAIL-induced apoptosis by up-regulating the expression of the death receptor 5 (Yoshida et al., 2007), and was also shown to sensitize refractory breast cancer cells to trastuzumab, a monoclonal antibody against HER2 (Rowe et al., 2008).

Precaution should be taken when using NDGA as anti-cancer agent, considering that, in particular types of cancer such as malignant glioma, NDGA can inhibit caspase 8 and 3, poly(ADP-ribose)polymerase cleavage and consequent CD95L-mediated apoptosis (Wagenknecht et al., 1998). Moreover, NDGA at low concentrations (< 0.3 µM) proved to moderately sustain the survival of some leukemic cancer cell lines, but becomes cytotoxic at higher, micromolar concentrations (Shaposhnikova et al., 2001). There is also evidence that LOX inhibitors like NDGA and baicailin can inhibit ferroptotic cell death caused by the accumulation of lipid-based ROS in acute lymphoblastic leukemia cells (Probst et al., 2017).

#### NDGA Inhibits Metastasis and Angiogenesis

NDGA impacts metastasis of tumor cells through LOX inhibition but also due to down-regulation of neuropilin 1, a single-pass transmembrane protein that functions as a “signaling platform” on the cell surface. Neuropilin 1 is over-expressed in breast, prostate, pancreatic, colon, and kidney cancers, and exerts important roles in tumor progression, angiogenesis and anti-cancer immunity (Rizzolio and Tamagnone, 2011). As shown in human PC3 prostate cells, decreased levels of neuropilin 1, induced by NDGA treatment, lead to alterations in the motility and cell-matrix adhesion, and attenuated tumor metastasis in a nude mice model of prostate cancer. Thus, neuropilin 1 suppression impacts on both tumor cells and the tumor microenvironment by down-regulating angiogenesis and extracellular matrix formation during the progression of metastasis (Li X. et al., 2016).

The formation of new capillaries from preexisting vessels is tightly regulated process that involves a complex network of cells, soluble factors and extracellular matrix molecules (Nie et al., 2000). Therefore, the persistently deregulated angiogenesis found in cancer is critically involved in promoting tumor growth and metastasis. Vascular endothelial cells express LOX and the resulting eicosanoids have potent biologic activities in these cells. This is probably the reason why NDGA can efficiently limit angiogenesis and hence tumor outgrowth. The pan-LOX inhibitor NDGA and the selective 12-LOX inhibitor baicalein, both exhibiting also antioxidant properties, were shown to reduce the expression of the vascular endothelial growth factor (VEGF) in human prostate cancer PC3 cells through inhibition of Sp1 (specificity protein 1) which is the transcription factor responsible for 12-LOX-mediated stimulation of VEGF (Nie et al., 2006). Moreover, proliferation and angiogenesis are suppressed by NDGA in breast cancer through inhibition of the rapamycin complex 1 (mTORC1), as demonstrated both in cultured breast cancer cells and in xenograft models (Zhang et al., 2012). NDGA reduced the basal level of mTORC1 and suppressed mTORC1 downstream signaling (expression of cyclin D1, hypoxia-inducible factor-α, and VEGF) by disrupting the mTOR/raptor interaction.

#### Controversies on the Antioxidant Activity of NDGA in Cancer Treatment

As described above, most of the anti-tumor effects of NDGA are supported by its inhibitory action on LOX enzymes and other inflammatory pathways as well as inhibition of receptor tyrosine kinase signaling pathways. Meanwhile, the stimulating action of NDGA on the cytoprotective transcription factor NRF2 raises concerns in oncologic pathologies (Milkovic et al., 2017). Various types of cancers (Kitamura and Motohashi, 2018) are characterized by chronic activation of the cytoprotective NRF2 system which accounts, at least partially, for the selection of more aggressive neoplastic phenotypes by conferring survival and growth advantage, as well as resistance to therapy (Cuadrado et al., 2018). Of utmost importance for cancer recurrence is the unique pattern of persistent NRF2 activation in cancer stem cells which sustains stemness and shields these cells against anti-cancer therapies (Kim et al., 2018). Therefore, activation of the NRF2 pathway by NDGA might be deleterious in advanced stages of cancer. From the antioxidant perspective, NDGA therapy seems to be in fact relevant for chemoprevention in patients at risk and in early steps of carcinogenesis, when persistent exposure of normal cells to oxidative stress and carcinogens may trigger their neoplastic transformation. In this case, NRF2-mediated transcription of cytoprotective genes helps to restore the redox balance, as well as to avoid unwanted DNA mutations and cancer initiation. Nevertheless, intensive research is nowadays ongoing for better defining the boundaries between NRF2 positive and negative effects in cancer, and for establishing a precise rationale for undertaking NRF2 therapeutic targeting (Milkovic et al., 2017). It is worth mentioning that NDGA might turn into a pro-oxidant at higher doses (Biswal et al., 2000; Sahu et al., 2006) which sustains apoptosis of tumor cells, hence supporting the use of NDGA in cancer treatment either as monotherapy or as adjuvant for conventional therapeutic strategies.

#### NDGA Protects Normal Tissues Against the Deleterious Action of Anti-Cancer Therapies

Mundhe et al. (2015) found that NDGA ameliorated cisplatin-induced nephrotoxicity and was even capable of increasing *in vivo* the anti-tumoral properties of cisplatin in a 7,12-dimethyl benz[a]anthracene (DMBA)-induced breast cancer rat model (Mundhe et al., 2015). Cisplatin is toxic for the kidneys because it promotes the generation of ROS and inhibits the activity of the endogenous antioxidant defense system. The protective action of NDGA was mirrored by the reduction in the levels of serum creatinine and BUN (Blood Urea Nitrogen) and also by the increase of superoxide dismutases protein levels in the breast cancer tissue.

## Adverse Effects of NDGA

Despite compelling preclinical evidence on the potential benefits of NDGA treatment in various pathologies, the major drawback for further clinical development is related to its important side-effects (Alderman et al., 1994; Lu et al., 2010). Most of the available information about safety issues in humans has been obtained from consumption of the chaparral infusion, which is a non-standardized mixture of compounds extracted from *Larrea*, in which NDGA is the main constituent and may generally contain uncontrolled carcinogens or tumor promoters (Gold and Slone, 2003; Bode and Dong, 2015). Nevertheless, preclinical data gathered with pure NDGA indicate that many of the toxic effects of chaparral tea can be attributed to this compound, particularly in kidney and liver damage.

Kidney toxicity associated with NDGA, leading to cystic nephropathy, was initially reported in rats (Goodman et al., 1970). Later on, a case report in humans further associated high consumption of chaparral tea with cystic renal disease and cystic adenocarcinoma of the kidney (Smith et al., 1994). The liver is also highly affected by high consumption of NDGA in the form of chaparral tea. Thus, prolonged consumption of this infusion for over 10 months led to severe non-viral hepatitis (Gordon et al., 1995). The effects can be attributed to NDGA since a later study in mice demonstrated that intraperitoneal administration of NDGA dose-dependently increases the levels alanine aminotransferase in serum (Lambert et al., 2002). The toxic effect may be related, at least in part, to the conversion of NDGA to its *ortho*-quinone form. Indeed, a study performed with clone-9 rat hepatocytes demonstrated that exposure to high concentrations of NDGA (up to 100 µM) caused lipid peroxidation, DNA double-strand breaks and cell death (Sahu et al., 2006). In addition to kidney and liver damage, other organs are also affected by prolonged or high NDGD dosing. A clinical trial addressing the pharmacokinetics and efficacy of NDGA in non-metastatic recurrent prostate cancer evidenced the following side-effects on 12 patients, at a dosage of 2000 mg/day given orally in three divided doses: diarrhea (12/12), fatigue (5/12), headache (4/12), abdominal distension, and nausea (3/12). Elevated levels of alanine aminotransferase (8/1), aspartate aminotransferase (6/12), bilirubin (3/12), and alkaline phosphatase (2/12) were registered (Friedlander et al., 2012).

Although, the reported toxic doses of NDGA in humans and experimental animals generally exceeded the traditional use of the plant (Arteaga et al., 2005), based on the evidence of hepatotoxicity and nephrotoxicity the US Food and Drug Administration (FDA) removed NDGA from the list of Generally Regarded As Safe (GRAS) compounds. Moreover, food containing any added NDGA is deemed to be adulterated in violation of the act based upon an order published by FDA in the Federal Register in April 11, 1968.

The toxicity related to NDGA is most likely due to the oxidation of the catechol rings to their corresponding quinones, leading to adduct formation in proteins and glutathione depletion (Billinsky et al., 2007; Jeong et al., 2017). Moreover, catechol quinones form depurinating DNA adducts and DNA double strand brakes, leading to cancer and other diseases (Cavalieri et al., 2002). Therefore, NDGA analogues with potentially low toxicity are being developed by protecting the catechol groups from oxidation to their *ortho*-quinone derivative.

## NDGA-Analogs as Novel Therapeutic Small Molecules

**Figure 4:**
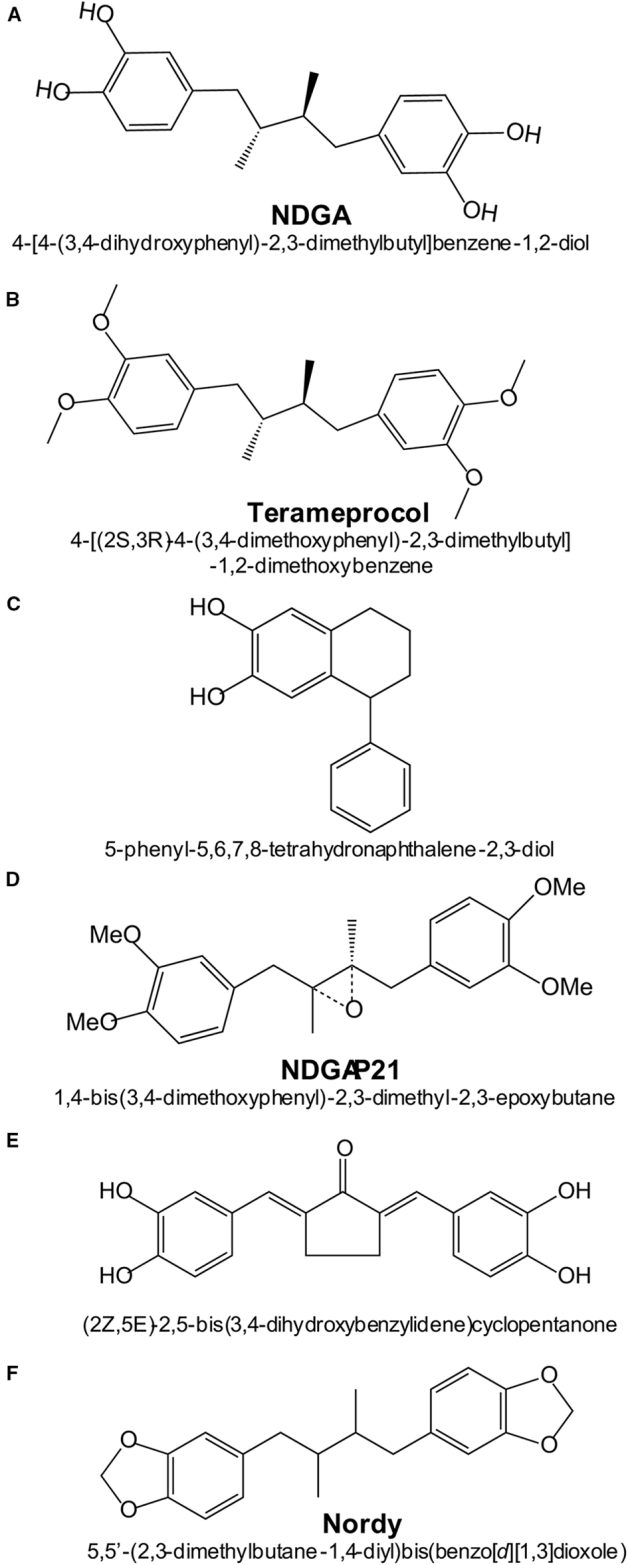
Chemical structures and names of NDGA and NDGA-analogs: **(A)** NDGA (PubChem ID 4534); **(B)** Terameprocol (PubChem ID 476861); **(C)** Re-draw of NDGA-analog reported in (Blecha et al., 2007); **(D)** Re-draw of NDGA-analog reported in (Zhao et al., 2017); **(E)** Re-draw of NDGA-analog reported in (Huang et al., 2018).

Several NDGA analogs are currently in various phases of development, aiming to increase therapeutic efficacy while limiting side-effects. **Figure 4** shows the most relevant NDGA analogs under clinical development.

Recently, a series of NDGA analogs with modified catechol rings were shown to correct metabolic alterations related to hepatic lipid metabolism in a high fructose diet-fed rat model of dyslipidemia, insulin resistance and hypertension (Singh et al., 2019). Oral gavage of these analogs reduced the hepatic and plasma levels of triacylglycerides. In particular, Nordy [(5,5-(2,3-dimethylbutane1,4-diyl)bis(benzo[*d*][1,3]dioxole); **Figure 4**] was very effective at inhibiting the expression of several genes involved in triacylglycerides synthesis (*Scd*, *Gpam* and *Dgat2*) and fatty acid elongation (*Elovl2* and *Elovl5*). These effects were consistent with inhibition of transcription factors SREBP-1c (sterol regulatory element binding transcription factor 1c) and ChREBP (carbohydrate-responsive element-binding protein). In contrast to NDGA, these analogues did not alter the expression of genes involved in hepatic fatty acid oxidation or transport.

In ischemic stroke, oxidative damage is a crucial factor. Novel NDGA analogs have been developed that combine potent ROS scavenging and NRF2 activation (Huang et al., 2018). **Compound 3a** of this study [(2Z, 5E)-2,5-bis (3,4-dihydroxybenzylidene)cyclopentanone; **Figure 4**] was more effective than the antioxidant edaravone in reducing brain infarction after cerebral ischemia-reperfusion injury in rats subjected to transient middle cerebral artery occlusion. However, this study did not compare the efficacy of this compound *vs.* NDGA in stroke protection or in overall toxicity and therefore, its benefit over NDGA remains to be ascertained.

High concentrations of NDGA are required to inhibit tumor growth, thus yielding toxicity. Therefore, an intensive search has been made to develop NDGA analogs with low toxicity. This is the case of tetra-O-methyl nordihydroguaiaretic acid, also called Terameprocol, M4N or EM-1421. As shown in **Figure 4**, in this molecule the four hydroxyl groups of the two catechol rings have been methylated, thus preventing the formation of catechol quinones. While NDGA intraperitoneally injected in mice has a LD_50_ value of 75 mg/kg, in a phase 1 clinical study it was found that Terameprocol is well-tolerated even at 1700 g/kg in humans (Chang et al., 2004). Terameprocol induces rapid cell death in combination with Etoposide and Rapamycin in prostate LNCaP cells, both *in vitro* and in animal experiments (Eads et al., 2009). Terameprocol entered Phase I/II clinical trials in patients with recurrent high grade lymphoma (Grossman et al., 2012) or advanced forms of leukemia (Tibes et al., 2015), but proved only low anti-tumor activity. Nevertheless, these clinical studies emphasized that Terameprocol has an improved toxicological profile as compared to NDGA and further reshaping could improve its anti-cancer activity. The pharmacological target appears to be the transcription factor Sp1 which controls a large number of genes, and therefore Terameprocol exerts pleiotropic effects: 1) it induces caspase-7 cleavage and inhibits autophagy by suppressing ATG5 and BNIP3; 2) it broadly modulates metabolic processes related to glucose-6-phosphate/glucose-1-phosphate/UDP-glucose; 3) it reduces glutathione levels by up-regulation of CHAC1, a key enzyme that affects the stress pathways; 4) it suppresses energy metabolism by inhibiting the mitochondrial electron transport system (like NDGA), along with the TCA cycle; 5) it induces oxidative stress by decreasing the content of glutathione and propionylcarnitine, a superoxide scavenger. Transcriptomic and metabolomic analysis using high-throughput screening methods (GC/LC-MS and deep RNA sequencing) revealed that Terameprocol is a global transcriptional repressor of genes that are dependent on the Sp1 transcription factor. Studies in glioblastoma primary cultures and cell lines have shown that Terameprocol, in combination with temozolomide (TMZ), down-regulates the expression of Cdk1 and survivin, while the survivin-2B variant was up-regulated (Castro-Gamero et al., 2013). In this study, Terameprocol decreased cell proliferation separately and synergistically with TMZ, enhanced the effects of radiotherapy, especially when associated with TMZ, induced apoptotic cell death, decreased the mitotic index and arrested the cell cycle mainly in the G2/M phase. Once again it was demonstrated that Terameprocol could be successfully used as co-therapy in various types of cancer.

Blecha *et al.* prepared a series of NDGA-analogs to be more potent and selective against MCF-7 breast cancer cells by targeting the IGF-1 receptor (IGF-1R) or 15-LOX (Blecha et al., 2007). The NDGA analogs consisted of introducing various substituents into one of the two catechol rings. One of the analogs (5-phenyl-5,6,7,8-tetrahydronaphthalene-2,3-diol) showed higher specificity for IGF-1R (Blecha et al., 2007). Zhao et al. demonstrated that the NDGA analog NDGA-P21 [1, 4-bis (3, 4-dimethoxy phenyl)-2, 3-dimethyl-2, 3-epoxy butane] was capable of inhibiting the *in vitro* proliferation of glioma cells and their stemness. Under the action of NDGA-P21, the cell cycle was arrested in the G0/G1 phase. However, NDGA-P21 has limited water solubility which is a major drawback for further clinical development (Zhao et al., 2017).

In a very interesting work, Asiamah et al. found that the oxidative cyclization of NDGA forms a dibenzocyclooctadiene, which may have therapeutic benefits (Asiamah et al., 2015). Certain NDGA-analogs may be more susceptible to cyclize. Only NDGA-analogs that have two catechols have the capacity to form dibenzocyclooctadienes. The formation of quinones may not be a necessary step for the formation of dibenzocyclooctadienes, and cyclization depends on radicals (Asiamah et al., 2015). Furthermore, in order to test the hypothesis of reactive intermediate metabolites of NDGA, Asiamah et al. (2015) synthesized catechol- and phenol-type analogs of NDGA, aiming to study the formation of quinone-types, and found that the phenol-type NDGA-analogs are probably safer for clinical applications. It was presumed that the formed quinone-type methide would depend on the substitution in the aromatic rings, but found no evidence that *para*-quinone methide was formed, thereby suggesting that the reactive intermediate metabolite of NDGA that is toxic for the liver is *ortho*-quinone methide (Asiamah and Krol, 2018). Altogether, compelling preclinical and clinical evidence highlighted that some of the newly designed NDGA analogs hold great promise as therapeutic agents for cancer treatment, either as monotherapy or in combination with conventional anti-cancer agents.

Regarding the role of NDGA-derivatives in neurodegenerative disease, Daniels et al. (2019) found that the cyclized NDGA analog prevented the aggregation of α-synuclein into amyloid-like fibrils by producing modified monomers of α-synuclein that are aggregation-resistant. Cyclized NDGA reduced neurodegeneration in a *Caenorhabditis elegans* α-synuclein-driven neurodegeneration model. The cyclized NDGA analog has to be capable of oxidation because this fact is critical for preventing α-synuclein aggregation (Daniels et al., 2019). Huang et al. (2018) synthesized NDGA-analogs containing curcumin’s α, β-unsaturated ketone moiety (Huang et al., 2018). The analogs provided cytoprotection against oxidative damage. One of the analogs [(2Z,5E)-2,5-bis(3,4-dihydroxybenzylidene] cyclopentanone) promoted NRF2 translocation to the nucleus and the expression of heme oxigenase-1 *in vitro* in PC12 cells (pheochromocytoma of the rat adrenal medulla). This analog was proposed for the treatment of cerebral ischemia-reperfusion injury in stroke, especially due to its lower cytotoxicity compared to other analogs, protection against hydrogen peroxide and reduction of lipid peroxidation markers such as MDA.

## Concluding Remarks

For over 60 years, preclinical studies in cell culture and rodents indicate that the lignan NDGA is a promising drug for prevention or therapy of several chronic diseases and cancer. However, very little progress has been made in the translation of these studies to a clinical setting. This fact may reflect the large variety of side effects described for NDGA. While NDGA might be considered as a multi-target small molecule that elicits anti-oxidant and anti-inflammatory responses at particular dose ranges, it is also clear that under many difficult-to-control conditions it may also produce harmful effects. In recent years a new impulse to this field has been given with the development of NDGA analogs that might be more potent and target-selective, and at the same time exhibit lower toxicity due to prevention of the catechol conversion into quinone. This is the case of Terameprocol, currently used in several clinical trials on cancer. Also, some NDGA analogs are promising in neurodegenerative disorders and in metabolic syndrome. However, much work needs to be done in order to define and attain a safe pharmacological profile of NDGA derivatives. Increasing the knowledge about their pharmacokinetics, pharmacodynamics and mechanisms of action will be crucial to translate findings from traditional medicine to official medicine.

## Author Contributions

GM wrote the section addressing lipoxygenases and cancer, and participated in writing of all sections. EM-K, JP-C, and AR wrote the sections on liver and kidney diseases. AR wrote the section neurodegenerative diseases. AC wrote the remaining sections and participated in writing of all sections.

## Funding

This work was supported by SAF2016-76520-R of the Spanish Ministry of Economy and Competiveness; P-024-FTPGB 2018 from the Spanish “Tatiana de Guzman el Bueno Foundation” and by the P_37_732/2016 grant (REDBRAIN) financed by the European Regional Development Fund, Competitiveness Operational Program 2014–2020. EM-K was financed by a Postdoctoral Scholarship from Consejo Nacional de Ciencia y Tecnología (CONACY, Mexico) with support from the Posgrado en Ciencias Biológicas at UNAM. Comunidad Autónoma de Madrid (grant B2017/BMD-3827).

## Conflict of Interest

The authors declare that the research was conducted in the absence of any commercial or financial relationships that could be construed as a potential conflict of interest.

## Abbreviations

4-VO, four-vessel occlusion; 5-HEPE, 5-hydoxyeicosapentaenoic acid; 5-HETE, 5-hydroxyeicosatetraenoic acid; AA, arachidonic acid; AD, Alzheimer’s disease; ALS, Amyotrophic Lateral Sclerosis; ARE, Antioxidant Response Element; ALT, alanine aminotransferase; AST, aspartate aminotransferase; beta-TrCP, beta-transducin repeat-containing protein; BTB, broad complex, Tramtrack, Bric-a-brac; bZip, basic region-leucine zipper; COX, cyclooxygenase; CUL3/RBX1, cullin 3 and RING-box protein 1; DGR, double glycine repeat; DHA, docosahexaenoic acid; DPP, dipeptidyl Peptidase 3; DUSPs, dual specificity phosphatases; EPA, eicosapentaenoic acid; EWL, egg white lysozyme; FAM117B, Family with sequence similarity 117 member B; GSH, glutathione; HD, Huntington’s disease; HO-1, heme oxygenase-1; HPETEs, hydroperoxy eicosatetraenoic acids; IGF-1R, insulin-like Growth Factor 1 Receptor; IGF-I, insulin-like growth factor-1; IKBKB, inhibitor of Nuclear Factor Kappa B Kinase Subunit Beta; IP-10, interferon-gamma inducible protein-10; IRF-1, interferon regulatory factor-1; KEAP1, Kelch-like erythroid cell–derived protein with Cap’n’collar homology (ECH)-associated protein 1; LDH, lactate dehydrogenase; LOXs, lipoxygenases; LTA4, leukotriene A4; LTB4, leukotriene B4; LTBR1/2, leukotriene B4 receptor 1 or 2; MAD2L1, mitotic Arrest Deficient 2 Like 1; MAF, small musculo aponeurotic fibrosarcoma proteins; MCAO, middle cerebral artery occlusion; MCC, mutated in colorectal cancers; MCMBP, minichromosome maintenance complex component; MCP-1, monocyte chemoattractant protein-1; MMP, matrix metalloproteinases; NDGA, nordihydroguaiaretic acid; NO, nitric oxide; NRF2, Nuclear factor erythroid 2-related factor 2; OGD, oxygen-glucose deprivation; PALB2, partner and localizer of BRCA2; PD, Parkinson’s disease; PGAM5, PGAM Family Member 5, Mitochondrial Serine/Threonine Protein Phosphatase; PI3K, phosphatidylinostisol 3-kinase; PKD, polycystic kidney disease; PPARγ, peroxisome proliferator-activated receptor gamma; PTEN, phosphatase and tensin homolog; PUFA, polyunsaturated fatty acids; ROS, reactive oxygen species; SLK, STE20 like kinase; Sp1, specificity protein 1; SQSTM1, Sequestosome 1; tBHQ, tert-Butylhydroquinone; TDP-43, TAR DNA binding protein 43; TGF-β, transforming growth factor β; TMZ, temozolomide.
